# Colloid chemistry pitfall for flow cytometric enumeration of viruses in water

**DOI:** 10.1016/j.wroa.2019.100025

**Published:** 2019-01-23

**Authors:** Elena A. Dlusskaya, Alexey M. Atrazhev, Nicholas J. Ashbolt

**Affiliations:** a School of Public Health, University of Alberta, Edmonton, Alberta, Canada; b Aquila Diagnostic Inc., Edmonton, Alberta, Canada; c Department of Medical Microbiology and Immunology, University of Alberta, Edmonton, Alberta, Canada

**Keywords:** Colloid, Auto-fluorescence, FCM, Virus enumeration, SYBR^®^ green I

## Abstract

Flow cytomtery (FCM) has become a standard approach to enumerate viruses in water research. However, the nature of the fluorescent signal in flow cytometric analysis of water samples and the mechanism of its formation, have not been addressed for bacteriophages expected in wastewaters. Here we assess the behaviour of fluorescent DNA-staining dyes in aqueous solutions, as well as sensitivity and accuracy of FCM for enumeration of DNA-stained model bacteriophages λ, P1, and T4. We demonstrate that in aqueous systems fluorescent dyes form a self-stabilized (pseudolyophilic) emulsion of auto-fluorescing colloid particles. Sample shaking and addition of surfactants enhance auto-fluorescence due to increased dispersion and, in the presence of surfactants, stabilization of the dye emulsion. Bacteriophages with genome sizes <100 kbp (i.e. λ & P1) did not generate a distinct population signal to be detected by one of the most sensitive FCM instruments available (BD LSR Fortessa™ X-20), whereas the larger T4 bacteriophage was resolved as a distinct population of events. These results indicate that the use of fluorescent dyes for bacteriophage enumeration by flow cytometry can produce false positive signals and lead to wrong estimation of total virus counts by misreporting colloid particles as virions, depending on instrument sensitivity.

## Highlights

• Fluorescent dyes form auto-fluorescent colloidal particles in aqueous solutions.
• Presence of surfactants enhances non-specific dye fluorescence.
• Successful virus enumeration relies on correctly identified target population.
• Stained virus samples demand gentle handling.

## Graphical abstract

**Figure:**
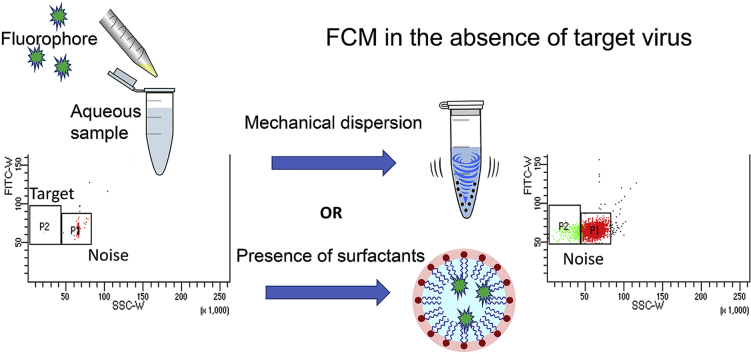

## 1 Introduction

Viruses are the most numerous microbial group and have a fundamental impact on aquatic ecosystem dynamics (Steward et al., 2013; Kauffman et al., 2018). They influence biogeochemical cycles through gene regulation and configuring microbial communities, and by “killing the winning” prokaryotic or eukaryotic species (Weinbauer and Rassoulzadegan, 2004), thereby maintaining the diversity and dynamic functioning of natural (Fauvel et al., 2017) and artificial (Withey et al., 2005) ecosystems. Key features include short-duration virus infection cycles, highly abundant viromes and rapid changes in species abundance and diversity.

To investigate viruses in environmental waters, transmission electron microscopy (TEM) was one of the first methods to be utilized, which demonstrated much higher abundances of viruses in marine waters compared to plaque forming unit enumeration (Bergh et al., 1989). With the development of sensitive fluorescent dyes, TEM was replaced by epifluorescent microscopy (EFM) (Noble and Fuhrman, 1998; Patel et al., 2007), which has demonstrated even higher counts, compared with TEM (Hermes and Suttle, 1995; Weinbauer and Suttle, 1997). Though sensitive, these direct methods are labor intensive and time consuming. Flow cytometry (FCM) enumeration of viruses has neither of these shortcomings, and was first reported in 1979 (Hercher et al., 1979), but was not widely used in ecological studies until twenty years later with the availability of bright fluorescent DNA-binding dyes. Since then, flow cytometric virus enumeration has become a standard approach in water research (Marie et al., 1999).

The efficiency of virus-targeted FCM is usually estimated by its comparison with TEM or EFM virus counts in environmental samples. To our knowledge only Tomaru and Nagasaki (2007) attempted to compare FCM counts with most probable number estimates, based on a culture and extinction dilution method (Suttle, 1993) using single virus cultures. In general, SYBR^®^ Green I is preferred for virus staining since this fluorescent dye is affordable and results in higher virus counts when compared to other dyes (Brussaard, 2004).

The aims of this study were to illustrate likely artifacts and understand their mechanisms when staining bacteriophages with SYBR^®^ Green I for FCM enumeration, and to estimate the sensitivity and accuracy of FCM for lambda (λ), P1, and T4 bacteriophage enumeration compared to PFU estimations.

## 2 Materials and methods

### 2.1 Bacteriophage sample preparation

Bacteriophages of three genome sizes: 48,502 bp dsDNA lambda (Sanger et al., 1982); 93,601 bp dsDNA P1 (Łobocka et al., 2004); and 168,903 bp dsDNA T4 (Miller et al., 2003) were propagated in *E. coli* hosts TG1 (Lucigen), MG1655 (ATCC 47076), and BL21DE3 (Sigma-Aldrich) respectively. The *E. coli* cultures were grown in LB broth (BD, REF# 241420) at 37 °C and 250 rpm to optical densities of 0.6–0.7, then infected with appropriate bacteriophage and the incubation was continued overnight at 37 °C with no shaking.

Overnight cultures were centrifuged at 4,000 *g* for 30 min to precipitate bacterial cell debris, supernatant was filtered through 0.22 μm syringe filter (Merck Millipore, REF # SLGS033SS) into a sterile Amicon Ultra 100 K centrifugal filter device (Merck Millipore, REF # UFC910024), and centrifuged again at 4,000 *g* for 20 min to eliminate any influence of growth media on flow cytometry analysis. Bacteriophage remaining on the filter part of the device (in about 250 μL), was treated with DNAse I (Roche Diagnostics, cat # 10104159001) to remove residual host DNA by adding: 25 μL of 10x DNAse I buffer (100 mM Tris HCl pH 7.5, 25 mM MgCl_2_, and 5 mM CaCl_2_ in MQ water) and 1 μL of 2.5 mg/mL DNAse I, dissolved in storage buffer (20% glycerol in 75 mM NaCl) to the bacteriophage suspensions and incubated for 45 min  at 37 °C. All chemicals were purchased from Sigma, unless stated otherwise.

After the incubation, bacteriophage samples were rinsed with 10 mL of 1x HyClone PBS (HyClone Laboratories, REF #SH30256.02) that was filtered through 1 kDa Macrosep Advance Centrifugal device (PALL, REF # MAP001C36), resuspended in PBS to the initial volume and analysed.

### 2.2 Bacteriophage double agar overlay plaque enumeration assay

Solid and soft Trypticase Soy Agar was prepared from BBL Trypticase Soy Broth (BD, REF # 211768) with addition of 1.5 and 0.6% agar respectively. Triplicate decimal dilutions of bacteriophage (T4, λ or P1) samples were prepared in 900 μL of 1x HyClone PBS and the double-layer agar assay was carried out as described previously (Kropinski et al., 2009). Standard deviations and P-values were calculated with Microsoft Excel™.

### 2.3 SYBR green I auto-fluorescence

The molecular structure of SYBR^®^ Green I (National Center for Biotechnology Information. PubChem Compound Database; CID = 10436340, https://pubchem.ncbi.nlm.nih.gov/compound/10436340 *(accessed July 20, 2017)*) implies a hydrophobic compound, which is not fully soluble in aqueous solvents. Hence, to estimate fluorescence of colloidal SYBR particles, we prepared stabilized emulsions of SYBR with each of the following surfactants: Triton-X100, IgePal-630, Tween 20, NP 40, Brije 35, and Sodium Dodecyl Sulfate (SDS). SYBR Green I (ThermoFisher, REF#S7563) was added to 1% solution of a surfactant in 1 kDa – filtered Tris-EDTA (TE) buffer pH 8.0 to final concentration of 50 x. All samples and SYBR Green I stock in this study were diluted, stained, and stored in black microcentrifuge tubes (Agros Technologies, REF# T7100BK).

Duplicate dilutions of SYBR in TE were prepared at 0.5x, 1x, 5x, and 50x concentrations; one set was heated at 80 °C for 10 min, and the other was analysed unheated. All TE buffer was 1 kDa – filtered before use. Crimson fluorescent 0.2 μm FluoroSpheres^®^ (ThermoFisher Scientific #F8806) were added to a final concentration of 3.4 × 10^7^ beads.mL^−1^ for quality control.

**Fig. 1:**
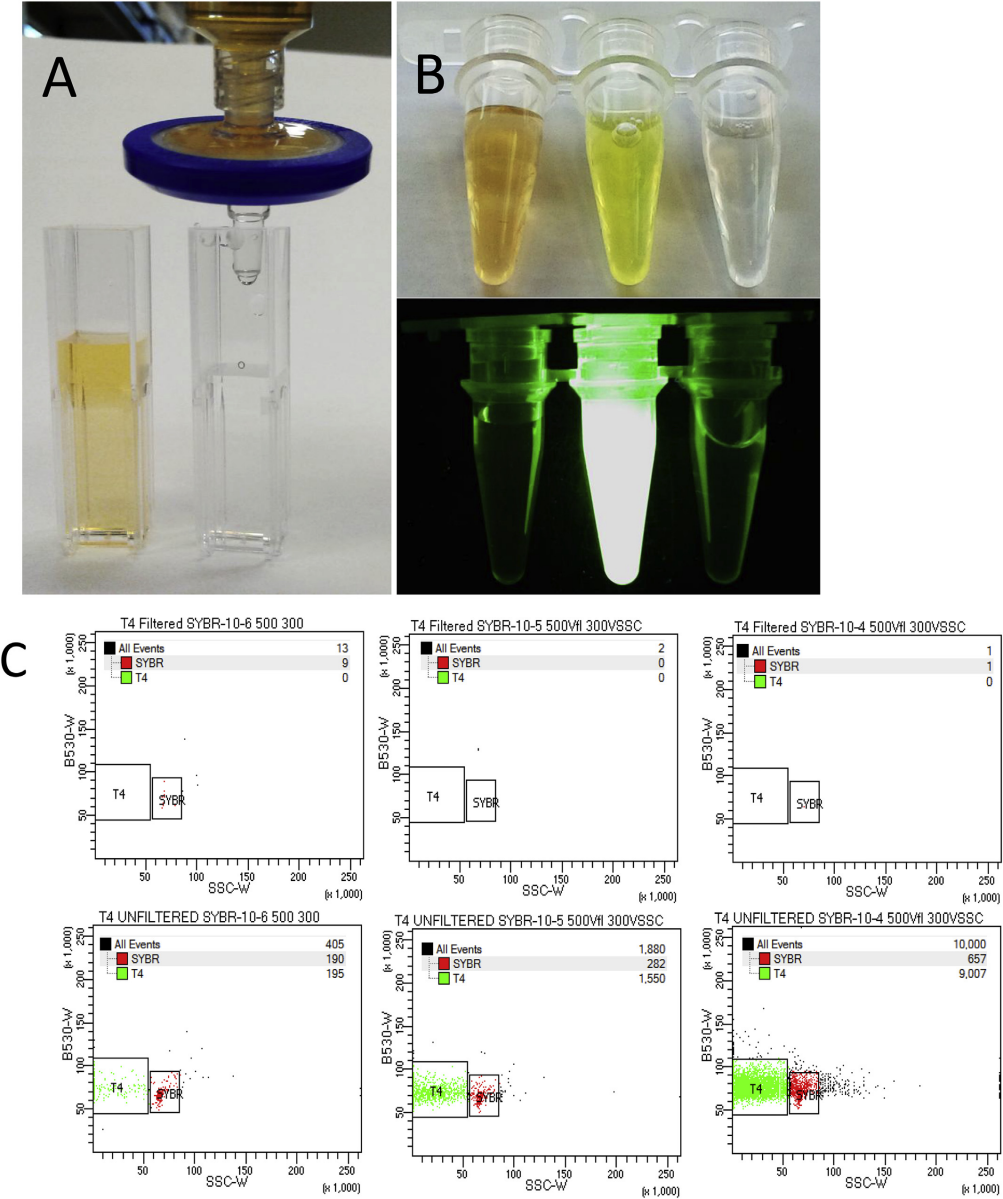
Removal of SYBR Green I from solution by coalescence on a membrane for aqueous solutions sterilization (EMD Millipore REF# s). 50x SYBR Green I in 1-kDa filtered 1x TE before (left) and after (right) filtration through 0.22 μm syringe filter.**A** Left to right: 25x unfiltered SYBR Green I in TE buffer; 25x unfiltered SYBR Green I and 150 ng μL^−1^ double stranded DNA in TE; 25x filtered SYBR Green I and 150 ng μL^−1^ double stranded DNA in TE. Visualised in natural light (top) and in UV (bottom).**B** Flow cytometric signal of bacteriophage T4 dilution series, stained with filtered (upper row) and unfiltered (lower row) SYBR Green I. (For interpretation of the references to colour in this figure legend, the reader is referred to the Web version of this article.)

The working stock of SYBR Green I should not be filtered due to interactions that remove this hydrophobic dye from solution (Fig. 1). This effect is based on well understood selective wettability and capillary force mechanisms in colloid systems (Yu et al., 2016).

Fluorescence was observed with a conventional benchtop UV transilluminator (UVP, ThermoFisher Scientific) as well as an EVOS FL fluorescent cell imaging system (ThermoFisher Scientific). For the wet mount, 25 μL of fresh samples were placed on new pre-cleaned microscope slides (ThermoFisher Scientific #12-550-A3) and glass coverslips (ThermoFisher Scientific #12-540B). The EVOS images were captured in TxRed (585/29 Ex 624/40 Em), GFP (470/22 Ex 510/42 Em), and TRANS channels and image overlays were created.

### 2.4 Flow cytometry

SYBR^®^ Green I samples were diluted in TE buffer to final concentrations 0.1x, 0.2x, 0.5x, 1x, and 2x, with one set heat treated and the other not, as described above.

Bacteriophage decimal dilutions were prepared in triplicate in TE buffer and stained as described (Brussaard, 2004) with 0.5x and 1x SYBR^®^ Green I. TE buffer was also prepared with the SYBR dye as negative control.

Flow rate was estimated with 1 μm latex bead FluoroSpheres^®^ (ThermoFisher, REF# F8823). The beads were first briefly vortexed and then bath-sonicated for 1 min as recommended by the manufacturer; noting that vortexing only gave inconsistent results (data not shown). Triplicate 100-fold serial dilutions were prepared to 10^−4^, and then decimally to 10^−6^, immediately after the sonication step. It is important to pay attention that no droplet was left on the outer side of the pipette tip. Dilutions, used for analysis, were briefly vortexed and sonicated again right before being analysed. As each batch of beads has a Certificate of Analysis with the number of beads per mL indicated, it was possible to calculate the number of beads per mL of the working dilution. To calculate the flow rate, the number of events in the bead population was divided by bead concentration in the working dilution. Flow rate was calculated each time samples were analysed.

Flow cytometric analysis was carried out with BD LSRFortessa™ X-20  cell analyzer (BD Biosciences, USA) equipped with 488 nm excitation laser with standard filter setup. The trigger was set on green fluorescence (FITC channel). Data was collected using FITC-W/SSC-W dot plots. Events were gated based on SYBR in TE samples with no virus and T4 SYBR-stained decimal dilutions.

Also, an older model of flow cytometer, Gallios™ (Beckman Coulter), also equipped with 488 nm excitation laser, was used to compare sensitivity of the two instruments. Data were collected as FL1 INT/FL2 INT and/or FL1 TOF/SSC TOF plot, with the same no virus and T4 SYBR-treated samples used on the BD LSRFortessa™.

## 3 Results

### 3.1 SYBR^®^ auto-fluorescence interference

**Fig. 2:**
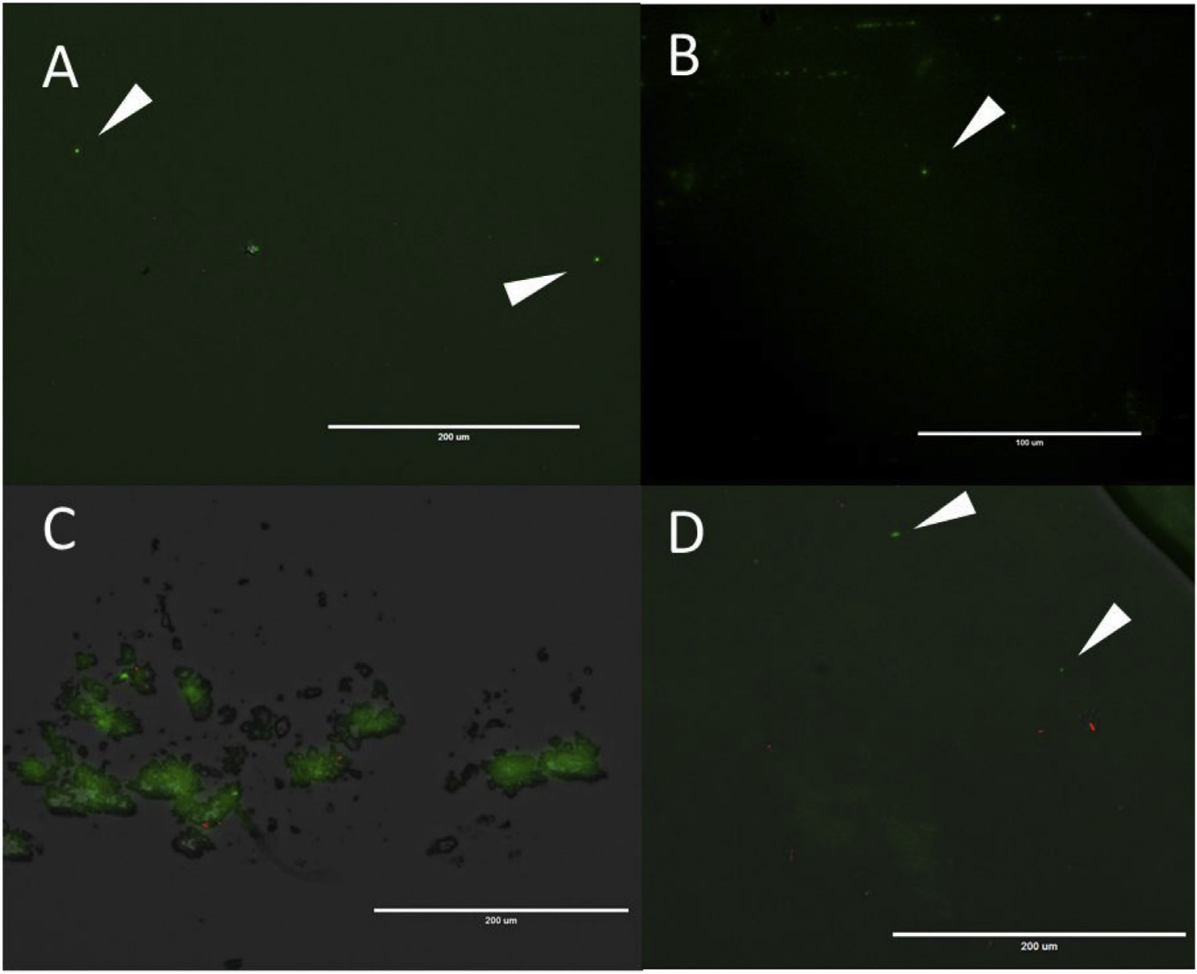
SYBR^®^ Green I by fluorescent microscopy in TE buffer: A) 0.5x; B) 1x; C) 2x; D) 5x. Red dots – 0.2 μm crimson FluoroSpheres^®^. Arrows indicate SYBR-colloid particles. (For interpretation of the references to colour in this figure legend, the reader is referred to the Web version of this article.)

Microscopic examination of SYBR^®^ Green I partly dissolved in TE buffer revealed the presence of fluorescent particles in all dilutions, in both heated and unheated samples. Critical to the presence of possible artifacts analysed by FCM, this dye produces small crystals or amorphous mass, which may also lead to uneven distribution of the SYBR fluorophore among the aliquots used for sample staining (Fig. 2). Centrifugation of SYBR stock is still not recommended as another well understood (Becher and Fishman, 1965) mechanical method for breaking an emulsion in addition to filtration.

**Fig. 3:**
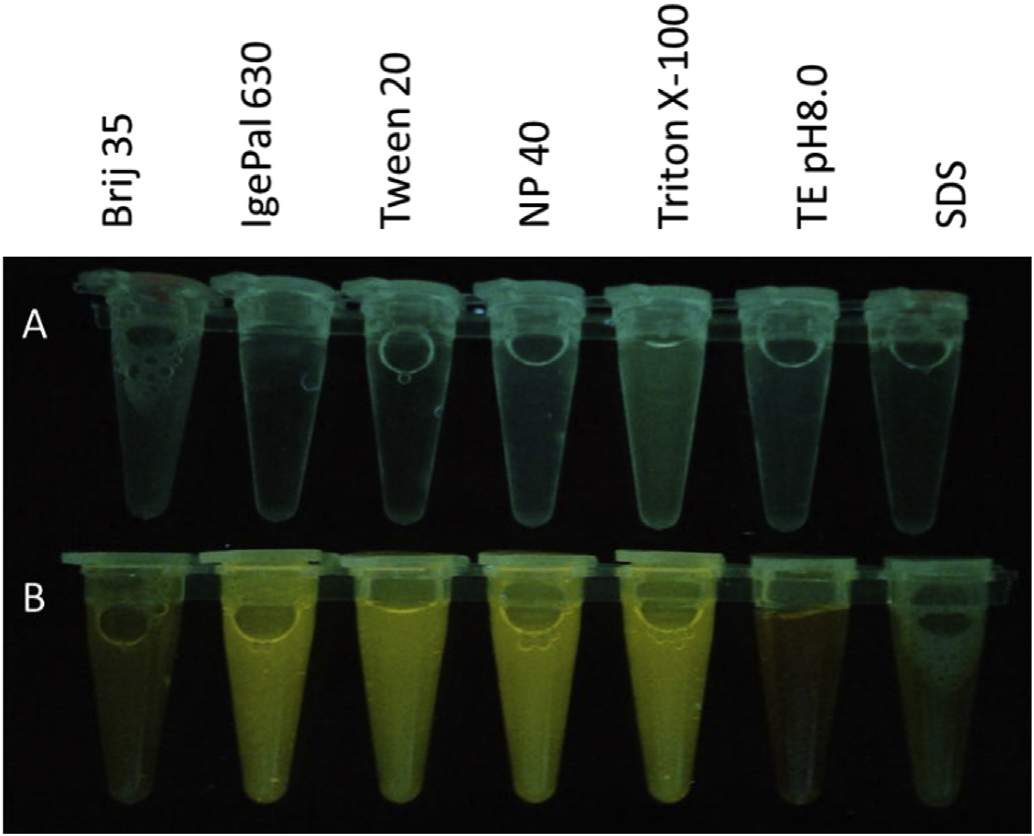
Fluorescence of SYBR^®^ Green I emulsified with various surfactants. A) 1% surfactants in 1 kDa-filtered TE pH 8.0; B) with 50x SYBR^®^ Green I added. (For interpretation of the references to colour in this figure legend, the reader is referred to the Web version of this article.)

**Fig. 4:**
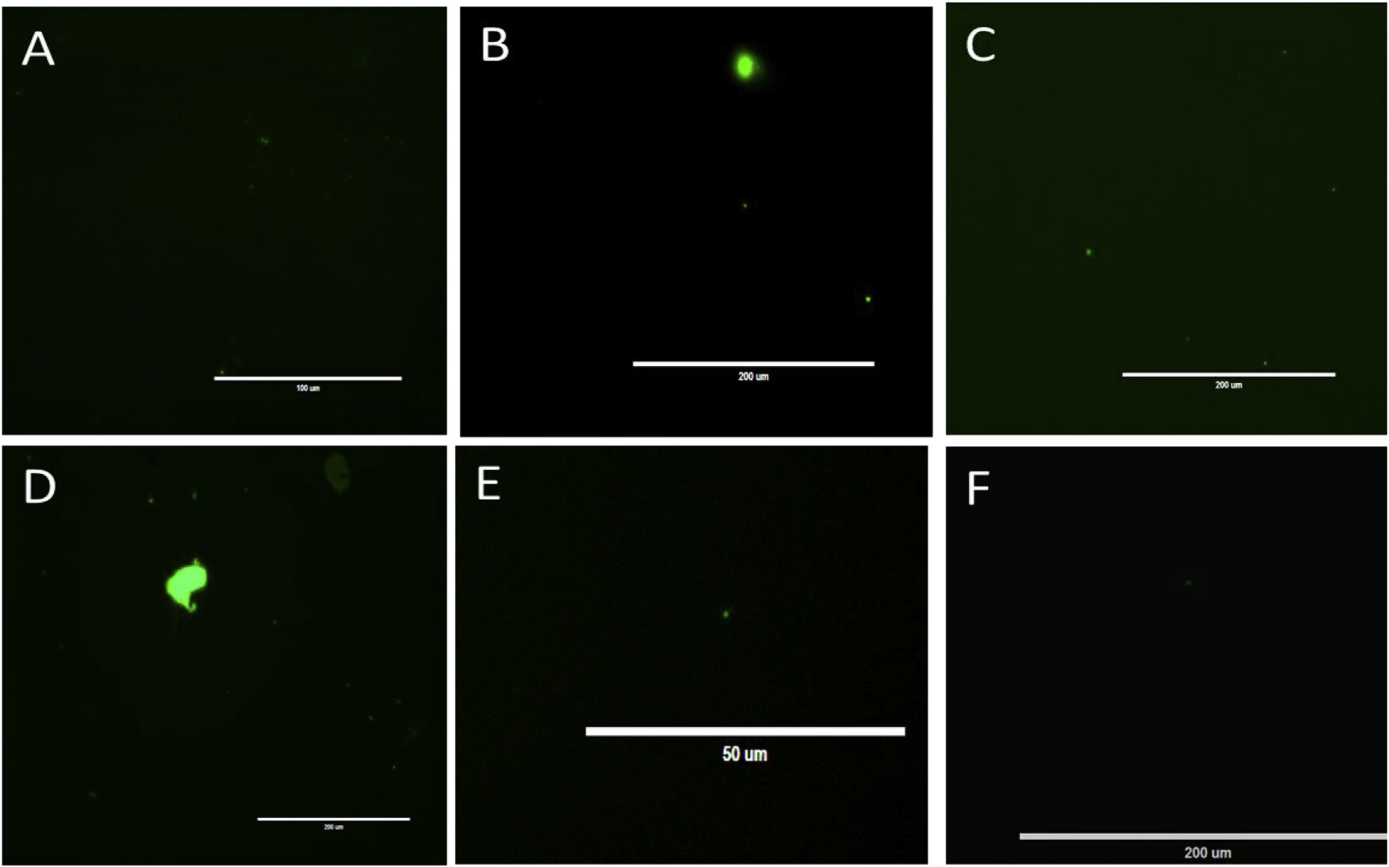
SYBR^®^ Green I emulsions by fluorescent microscopy, prepared with: A) BRIJ 35; B) Tween 20; C) NP 40; D) Triton-X100; E) EGEPAL CA-630; F) SDS. (For interpretation of the references to colour in this figure legend, the reader is referred to the Web version of this article.)

Addition of surfactants to 1% final concentration to aid colloid dispersion (relevant to maximum levels expected in wastewater (Adak et al., 2005)) resulted in intense fluorescence of SYBR^®^ Green I (Fig. 3) even with no DNA present. Similar results were obtained with SYBR Gold (ThermoFisher, REF#S11494) at 50x final concentration, Hoechst 33342 Ready Flow Reagent (ThermoFisher, REF#R17753) at 10% of commercial stock concentration, and some other fluorescent dyes (S1). Numerous fluorescing SYBR^®^ Green I particles were observed by microscopy (Fig. 4), and FCM signal was also more intense when compared to controls with no surfactant added.

**Fig. 5:**
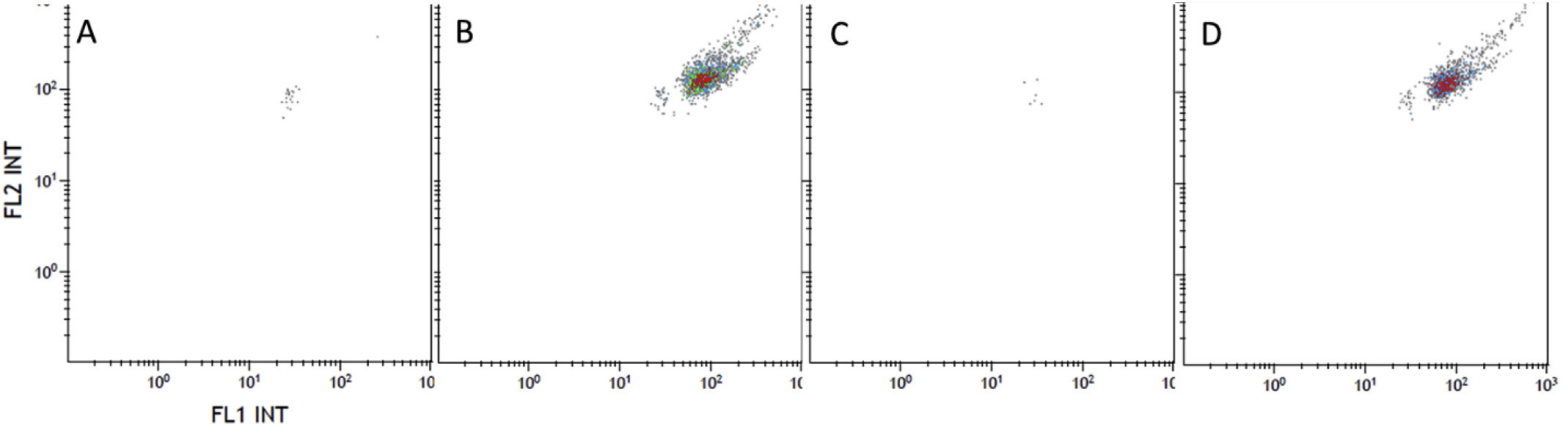
Fluorescent signal artifact of SYBR^®^ Green I-stained T4 bacteriophage sample (∼10^6^ PFU mL^−1^), obtained by GALLIOS™ flow cytometer. Measurements were performed in the same tube: sample gently transferred into FCM tube after staining (A), immediately after vigorous hand-shaking (B), 20 min after hand-shaking (C), after shaking second time (D). (For interpretation of the references to colour in this figure legend, the reader is referred to the Web version of this article.)

**Fig. 6:**
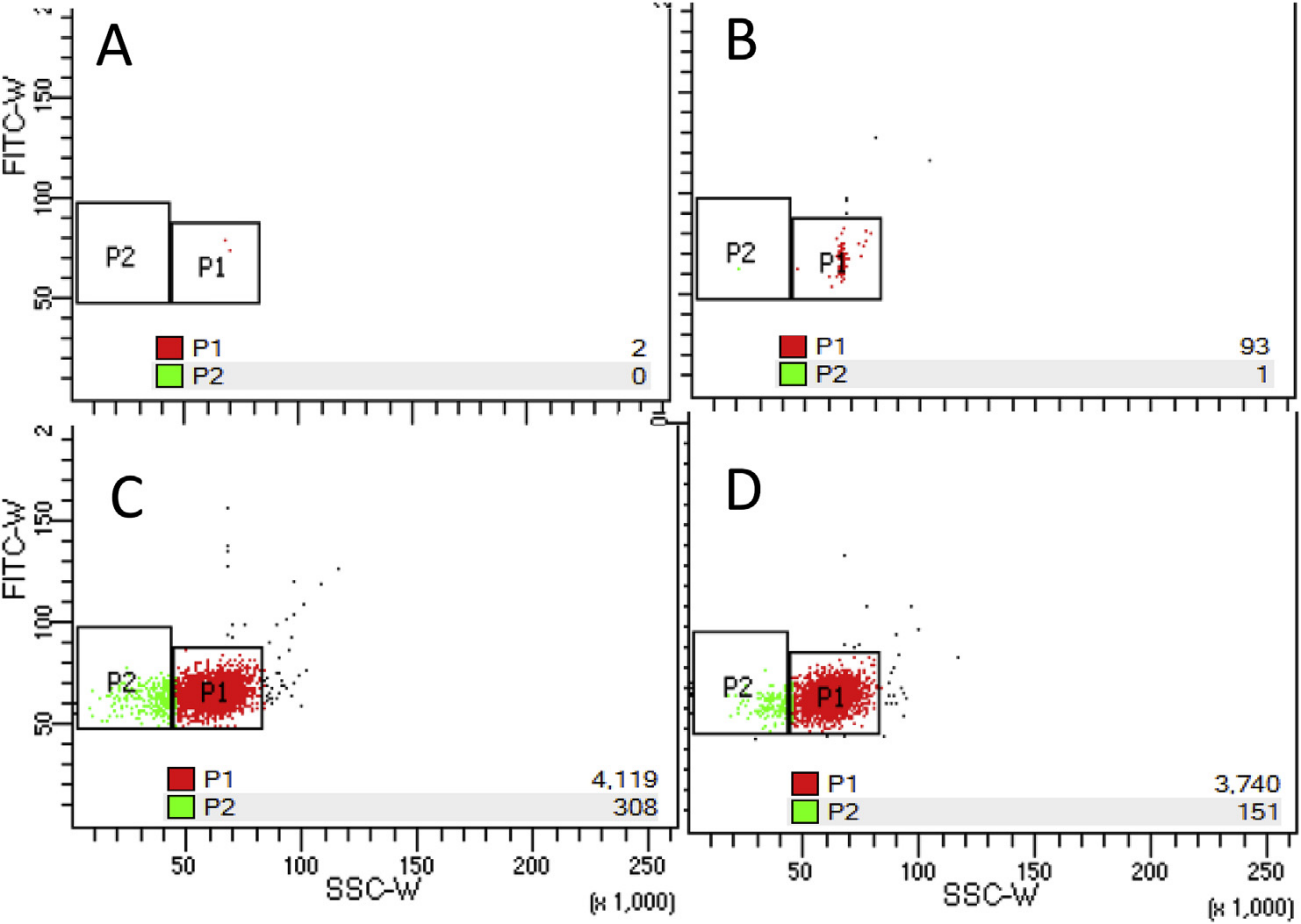
FCM fluorescent signal of 0.5x SYBR^®^ Green I in TE. No SYBR TE control (A), gently handled sample (B), and the same sample immediately and 10 min after vigorous pipetting (C and D). P1 – gated SYBR particle population, P2 – gate based on T4 virus signal. Immediately after pipetting SYBR population overlapped with the P2 gate. (For interpretation of the references to colour in this figure legend, the reader is referred to the Web version of this article.)

Flow cytometric analysis of various concentrations of SYBR^®^ Green I in TE demonstrated a distinct population of fluorescent particles. Event counts in some random sample tubes were much higher than in other replicate tubes with supposedly the same concentration of SYBR (data not shown). Most likely, this variability was the effect of non-uniform dispersion of SYBR^®^ Green I in the stock solution. Moreover, the event counts noticeably increased after bath-sonication, pipetting, or just hand shaking of the samples and decreased in the samples subsequently kept undisturbed, as illustrated in Fig. 5 when using the Gallios™ instrument and on the BD LSR Fortessa™ X-20 (see Fig. 6).

### 3.2 Bacteriophage detection and enumeration

**Fig. 7:**
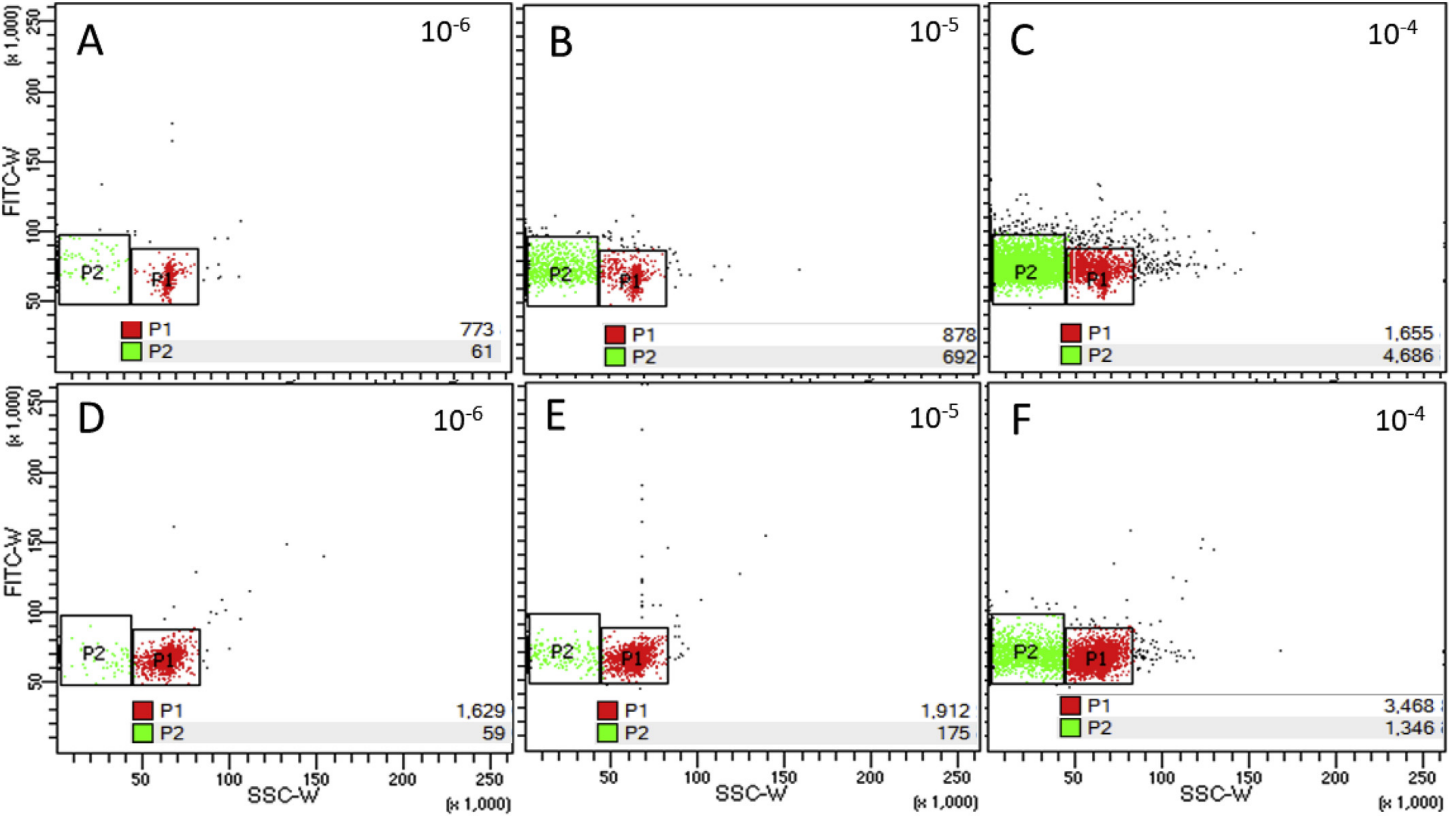
Flow cytometric analysis of SYBR^®^ Green I-labeled bacteriophage T4 at indicated decimal dilutions (population P2) (A–C). D–F: the same samples after vigorous pipetting. (For interpretation of the references to colour in this figure legend, the reader is referred to the Web version of this article.)

**Fig. 8:**
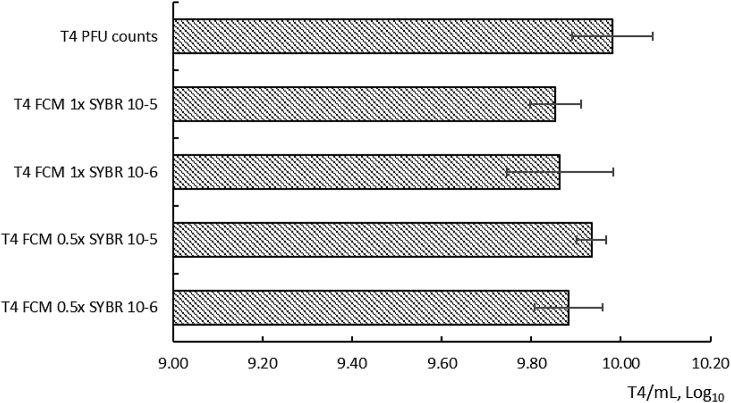
T4 virus enumeration by double agar overlay plaque assay (PFU/mL) and FCM using SYBR^®^ Green I labelling (N = 6, error bar is 1 SD).

Double agar overlay plaque assay showed 9.98 ± 0.09 log PFU.mL^−1^ of T4, 10.36 ± 0.25 log PFU.mL^−1^ of P1, and 9.3 ± 0.15 log PFU.mL^−1^ of λ bacteriophages. However, both Fortessa™ X-20 and Gallios™ instruments failed to detect Lambda (data not shown) and P1 (S2, S2a) bacteriophages. On the other hand, bacteriophage T4 was resolved as a distinct population of events when analysed on the Fortessa™ X-20 (Fig. 7, A-C; S3), but not with the Gallios instrument (Fig. 5). Two distinct populations were identified (P1 & P2), with only the number of events in P2 changing according to dilutions of the T4 bacteriophage, thus confirming P2 largely contained the target population. T4 bacteriophage FCM counts of the same bacterial lysate showed no significant difference (by two-tailed unpaired T-test, P > 0.05) between either 0.5x or 1x SYBR stained samples at either 10^−5^ or 10^−6^ dilutions, as well as when compared with the plaque assay counts (Fig. 8). However, significant disturbing of the samples led to decreased FCM virus events (Fig. 7, D-F), and estimated numbers did not correspond to the plaque assay data. Therefore, care in sample handling is also important when quantifying (T4) bacteriophages by FCM.

## 4 Discussion

### 4.1 FCM artifacts due to colloidal fluorophore particulates

As SYBR^®^ Green I is a hydrophobic chemical with low solubility in aqueous solvents (National Center for Biotechnology Information. PubChem Compound Database; CID = 10436340, https://pubchem.ncbi.nlm.nih.gov/compound/10436340 *(accessed July 20, 2017)*) there are inherent problems in using such fluorophores when targeting small particles like viruses by FCM. Though not widely discussed in the microbiological literature, SYBR^®^ Green I forms a disperse colloid rather than a homogenous molecular solution. Disperse systems can be formed via two main routes: mechanical dispersion or condensation from oversaturated solutions (Shchukin et al., 2001b, Shchukin et al., 2001a). For example, heating samples to 80 °C during staining procedure enhances oversaturation of the solution, and colloid particles start forming as the temperature decreases. The fact that fluorescent particles appear in both heated and unheated samples demonstrates that either route, or both routes together, might contribute to SYBR^®^ Green I dispersion.

A further indication of this dispersion was seen by the SYBR-FCM signal increase by shaking, sonication or pipetting, presumably due to the increase in auto-fluorescing dye colloidal particles. To the best of our knowledge, this is the first report of such chemical aspect of fluorescent dyes and its associated interference with small-particle enumeration by FCM. Consequently, keeping samples undisturbed for certain amounts of time reduces these apparent ‘virus’ event counts. This is a typical behaviour of lyophobic disperse systems (Shchukin et al., 2001b, Shchukin et al., 2001a). In such systems, mechanically dispersed colloid particles tend to coagulate, and if interaction energy between the particles allows, they will coalesce into larger particles. When the interaction energy is insufficient, due to low concentration and small diameter of remaining particles, coalescence becomes impossible and the disperse system self-stabilizes (Shchukin et al., 2001b, Shchukin et al., 2001a). Our findings demonstrate that SYBR^®^ Green I, as it is used in flow cytometry for virus enumeration, looks like a good example of a self-stabilized or pseudolyophilic system.

Such behaviour is not unique to SYBR^®^ Green I, as the fluorescent dye SafeView Plus™ (Applied Biological Materials Inc., REF #G468) was also shown to self-stabilize in solution in the same manner, which we confirmed by flow cytometry (S4). Furthermore, the addition of surfactants to a panel of SYBR^®^ Green I solutions generated and stabilized artifact particles into emulsions (Fig. 3), which could be misidentified as virus populations by FCM. Hence, when high gain levels are used to enumerate small-particle virions by FCM, hydrophobic fluorophores may generate various levels of false positive ‘virus’ signals. The same phenomenon was observed earlier by Pollard (2012), who compared the excitation and emission spectra of organic matter in water, in parallel with intact virus particles, and confirmed that about 70% of the fluorescent signal was associated with the matrix itself independently of the presence or absence of virus. Although Pollard did not use flow cytometry, his findings contribute to our observations that fluorescent colloid dye particles, present in dye-stained virus suspensions, can comprise a significant portion of the FCM signal.

Hence, the use of fluorescent dyes for virus enumeration by flow cytometry may produce false-positive signals and lead to overestimation of total virus counts by misreporting colloid particles as virions, depending on instrument sensitivity. Further research is needed to optimize reporting procedures involving small-particle count in pseudolyophilic colloid systems, so as to address stained-virus and no-virus but stain-present controls as discussed below.

### 4.2 Precautions for identification of target virion populations by FCM

To reduce misidentification of virions in environmental matrices, the instrument and assay sensitivity could be estimated using a panel of bacteriophages of various genome sizes. As such, the target population(s) could be identified by gating it/them from the total stained suspension signal. As illustrated in the current work, serial dilutions of the sample need to be correlated with the decline in target signal, which should be independent of dye concentration and should appear as a defined target population (e.g. Fig. 7). Once the population is identified and gated, FCM signal counts should correlate to bacteriophage enumeration by a second established method, such as culture-based plaque assay (e.g. Fig. 8). Stained no-virus aqueous phase control should always be applied during target identification, in order to minimize false-positive signals.

In addition, staining of virus particles with nucleic acid stains may require heating of the samples to 80 °C, in order to expose viral nucleic acid (Brussaard, 2009). Successful enumeration of nucleic acid targets relies on gentle handling of such heated samples. We speculate that in order for the number of fluorescent signals to correlate to the number of target nucleic acid molecules associated with virions, the freshly-heated and released viral DNA needs to remain compact. Rough handling of the sample could untangle the DNA molecule, creating distant contact points with the dye, and therefore decreasing the intensity of dye signal associated with a single DNA molecule.

## 5 Conclusions

Commonly used fluorescent dyes create pseudolyophilic colloid systems, which auto-fluoresce as stained virus-like particles even in the absence of DNA. The presence of surfactants further enhances non-specific fluorescence of such dye colloids and, therefore, use of surfactants for sample preparation should be avoided. Altogether, these interfere with small-particle enumeration by fluorescence-based assays, such as flow cytometry.

Successful enumeration relies on correct identification of the target population by the careful use of negative virus control samples. The instrument sensitivity should be assessed by comparison with established culture-based methods.

Given the pseudolyophilic colloidal nature of fluorophores used in FCM, sample handling can additionally affect the accuracy of virus enumeration. Overall, further research is needed to optimize the use of fluorescent dyes for virus quantification from environmental matrices by sensitive assays, such as flow cytometry.

## Conflict of interest

The authors have no conflict of interest to declare.
