# Supplementary material for: Colloid chemistry pitfall for flow cytometric enumeration of viruses in water
Source: Water Res X. 2019 Jan 23;2:100025. doi: 10.1016/j.wroa.2019.100025 (PMC6549941; doi:10.1016/j.wroa.2019.100025)
Supplement: Multimedia component 1 [file mmc1.pptx]

## Slide 1
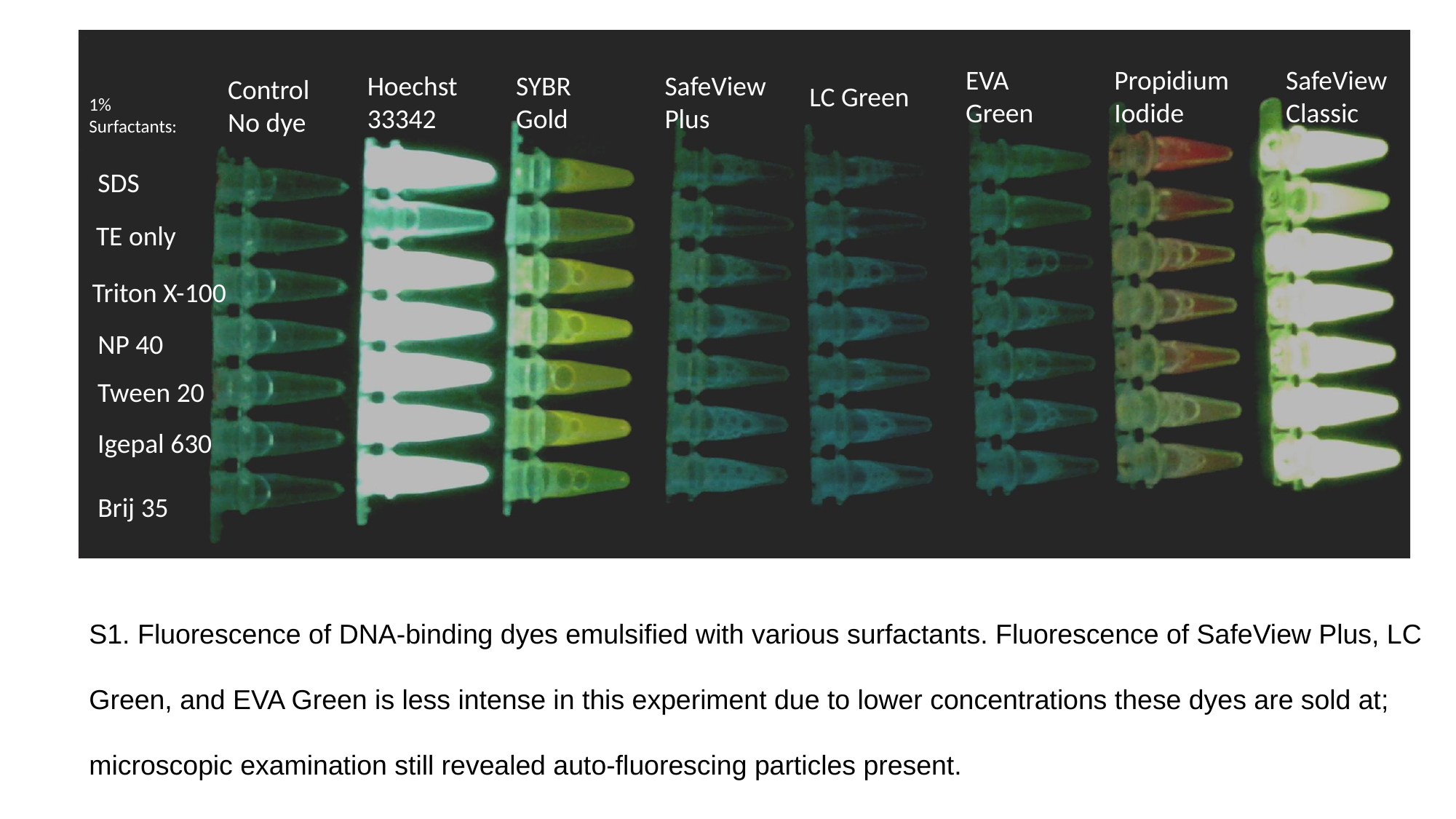

SafeView Classic
Propidium Iodide
EVA Green
Hoechst 33342
SYBR Gold
SafeView Plus
Control
No dye
LC Green
1% Surfactants:
SDS
TE only
Triton X-100
NP 40
Tween 20
Igepal 630
Brij 35
S1. Fluorescence of DNA-binding dyes emulsified with various surfactants. Fluorescence of SafeView Plus, LC Green, and EVA Green is less intense in this experiment due to lower concentrations these dyes are sold at; microscopic examination still revealed auto-fluorescing particles present.
