# Supplementary material for: Colloid chemistry pitfall for flow cytometric enumeration of viruses in water
Source: Water Res X. 2019 Jan 23;2:100025. doi: 10.1016/j.wroa.2019.100025 (PMC6549941; doi:10.1016/j.wroa.2019.100025)
Supplement: Multimedia component 3 [file mmc3.pptx]

## Slide 1
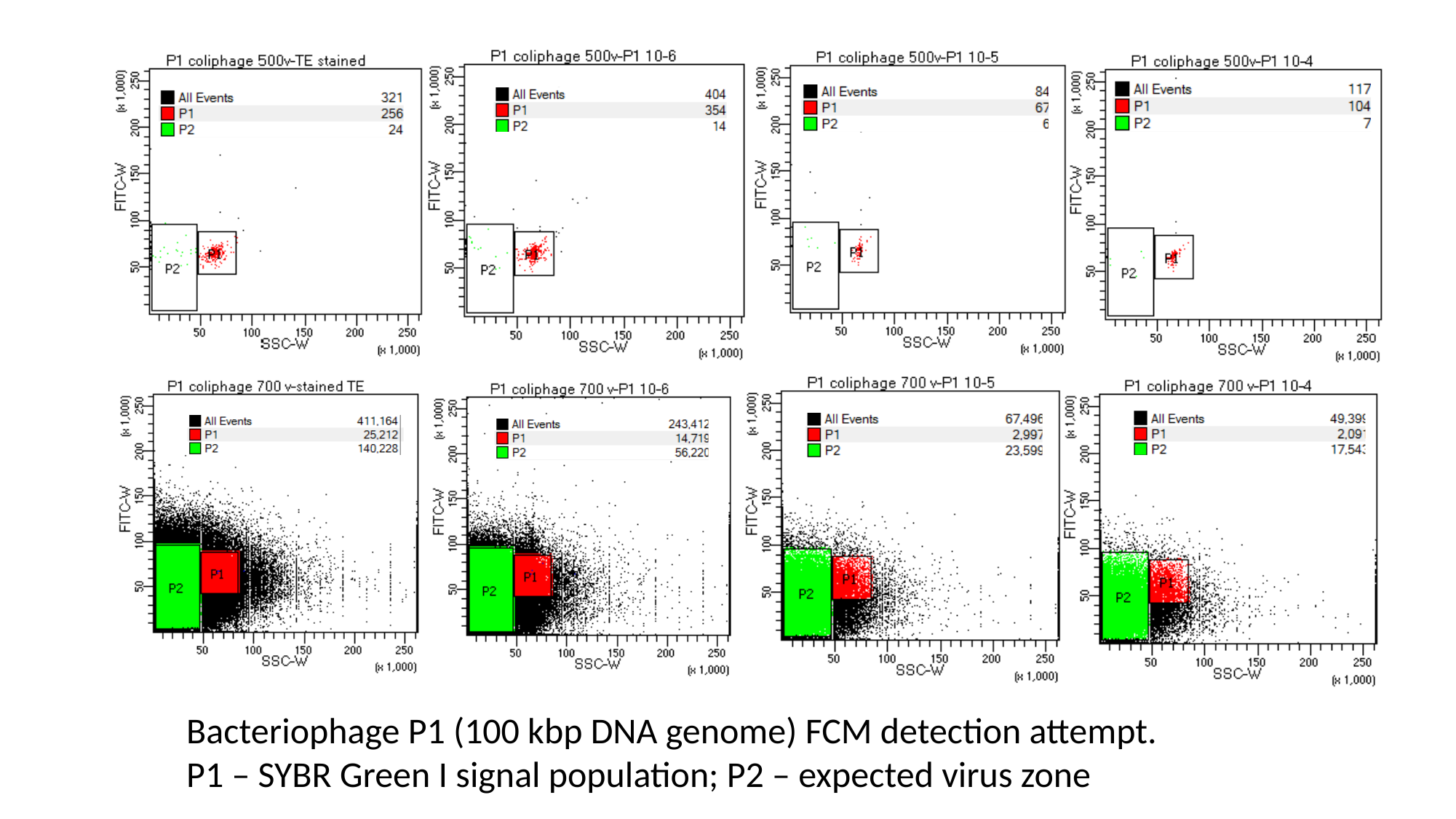

Bacteriophage P1 (100 kbp DNA genome) FCM detection attempt.
P1 – SYBR Green I signal population; P2 – expected virus zone
